# A study on depression of the elderly with different sleep quality in pension institutions in Northeastern China

**DOI:** 10.1186/s12877-020-01777-4

**Published:** 2020-09-29

**Authors:** Jun Zhang, Yingying Zhang, Zhenggang Luan, Xiujie Zhang, Haoran Jiang, Aiping Wang

**Affiliations:** 1 Nursing Department, The First Affiliated Hospital of China Medical University, 155 Nanjing North Street, Shenyang, 110001 China; 2 China Medical University, Shenyang, 110122 China; 3 Department of Intensive Care Unit, The First Affiliated Hospital of China Medical University, Shenyang, 110001 China

**Keywords:** Elderly, Pension institutions, Quality of sleep, Depressive symptoms, Influencing factors

## Abstract

**Background:**

China owns he largest aged population in the world, and the elderly adults who live in pension institutions are more likely to suffer from mental disorders than other elderly adults. The purpose of this study is to discover the risky factors of depression among nursing home residents with various sleeping quality.

**Methods:**

We conducted a cross-sectional study in Northeastern China from May to September in 2017 using multistage sampling method and 507 elderly people without cognitive impairment in six pension institutions were interviewed. The Pittsburgh Sleep Quality Index (PSQI) and Geriatric Depression Scale (GDS) were adopted to collect the information of sleep quality and depression. We used logistic regression to analyze the factors influencing depression among the elderly adults with poor or good sleep quality.

**Results:**

The overall depression rate among elderly adults was 21.7%. The logistic regression analysis revealed that marital status, chronic disease, regular exercise, physical ache, filial piety and chewing ability had significant effects on the depression of the elderly with good sleep quality. Loneliness, self-caring ability, chewing ability and chronic diseases had significant effects on depression of the elderly with poor sleep quality.

**Conclusion:**

The prevalence of depressive symptoms in the elderly is not high. However, sleeping quality distinguishes root causes on elderly adults depression. Therefore, the risk factors of depression among elderly adults should be analyzed separately.

## Background

Population aging is becoming an increasingly serious issue globally, posing serious challenges to the maintenance and/or improvement of human health and socioeconomic development, particularly in China [1]. China has the largest aged population and it is the only country in the world with over 100 million elderly adults (aged 60 years and above) [2]. With aging population and family re-structures, traditional home-based care in China has gradually weakened, and an increasing number of elderly people are inclined to receive long-term care in pension institutions [1]. The pension institutions in Northeastern China include public institutions and private institutions which can be found on government websites. The elderly can choose whether to stay in the pension institution voluntarily. Elderly adults who live in pension institutions rather than in their own homes are unable to receive care from their grown up children. Under such circumstance, they are more likely to suffer from mental disorders than other elderly adults.

Depression is one of the most important indicator of mental health. Among elderly adults, depression has become the second largest mental disorder after Alzheimer’s disease [3]. It is also the only mental disorder that ranks among the top in terms of disease burden in both high and low income countries (high-income countries: rank 2; low and middle-income countries: rank 4) [4]. It has been reported that about 80% of elderly adults who commit suicide as a result of depression [4]. Therefore, it is imperative to analyze depression among elderly adults living in pension institutions.

Sleep is a physiological need of the human body. Numerous studies have shown that the quality of sleep among the elderly is closely related to mental disorders, such as depression and anxiety [5, 6]. Elderly adults’ sleep quality certainly affects their lives, work, and mental status to varying degrees, and as such affects their quality of life. Therefore, it is necessary to conduct targeted research on the characteristics of the elderly population with different sleep quality. To date, there have been some studies on the relationship between sleep and depression among elderly adults. However, most of them are focusing on the correlation between sleep and depression among elderly adults who are either hospitalized or reside in a community. And yet there are few studies on elderly adults who are living in pension institutions [5].

In fact, previous studies in China have relied on different methods of measuring depression [7, 8], making it difficult to compare elderly adults depression levels on the same page. Moreover, researching conclusions from developed countries cannot be directly applied to situation in China because of differences in race, culture, income level, and lifestyle. Most prior studies also did not screen for cognitive impairment status among target group, leading to insufficient reduction of information bias and decreasing the generalization of previous conclusions [9].

This study attempted to analyze depression and factors influencing it among elderly adults with different sleep quality living in pension institutions in Northeastern China. We used the Geriatric Depression Scale (GDS), which is widely used within China and worldwide, to investigate depression among elderly adults living in pension institutions. Furthermore, we comprehensively analyzed and compared the depression levels among elderly adults according to their sleep quality and examined their correlation with demographic information and health status. Through this study, we provide rational prevention and intervention measures for elderly adults with different sleep quality that were suffering from depression.

## Methods

### Study area and subjects

This cross-sectional study was performed in Northeastern China from May to September 2017. First, according to the information of pension institutions on the website of the Civil Affairs Bureau, the institutions with more than 500 beds were selected. Liaoning is a province with 14 prefecture-level cities, however not too many pension institutions are qualified for this research. And mostly are located within and near the provincial capital. With the support for project funding, we selected eligible institutions from Shenyang, Anshang, Tieling and Benxi and trained the personnel accordingly. In consideration of the economic status, cultural background and cooperation level, we selected six pension institutions from these cities. Two in Shenyang, two in Anshan, one in Tieling, and one in Benxi. The inclusion criteria of the study population were: age ≥ 60; stay time ≥ 6 months; not diagnosed with Alzheimer’s disease; voluntary participation in the study. Exclusion criteria of the study population: Elderly with acute diseases during the investigation. We primarily double confirm with elder people’s family if elders have been diagnosed as Alzheimer as there was limited resources for us to look through. Besides, all the participants were interviewed by using a questionnaire exploring cognitive function, demographic characteristics, and influencing factors. The questionnaire also made based on the Pittsburgh Sleep Quality Index (PSQI) and Geriatric Depression Scale (GDS). The selection of sample size is 5–10 times of the number of items of all scales. The sample size needs to consider 10–20% error [10]. There were 52 items in this study. Therefore, the sample size should be 286 ~ 624. The sample size was proportionally determined based on each facility’s bed quantities. And participants were selected randomly. The specific sampling results is shown in Table 1. Considering the economic and human factors, the final sample size were 588. We contacted a total of 588 elderly people, of whom 553 were interviewed and completed questionnaires. After screening for the cognitive function using the Mini-Mental State Examination (MMSE) [11]. The MMSE has been widely used to screen for cognitive defects among the elderly10. The total score of the MMSE ranges from 0 to 30. Combined with the educational level of the elderly, the diagnostic criteria for cognitive impairment among the elderly are as follows: illiteracy is ≤17 points, primary school is ≤20 points, secondary school (including technical secondary school) is ≤22 points, and university is ≤23 points. Five hundred seven elderly adults without cognitive defects were chosen as participants.

**Table 1:** Sampling results of the elderly in pension institutions

City	Shenyang(1)	Shenyang(2)	Anshan(1)	Anshan(2)	Tieling	Benxi	Total
Number of beds	696	624	576	564	552	516	3528
Number of people selected	116	104	96	94	92	86	588

## Ethics approval and consent to participate

All participants were asked to fulfill a consent form prior participating the study. Each participants was well noted that they have the right to decline or withdraw from the survey at any time. Project detail has been introduced in detail with contact persons from each institution. And the project has been conducted with the permission from The First Affiliated Hospital of China Medical University (Address of: No.155, Nanjing Road, Shenyang 110001, China) under the protocol and consent approved by the Ethics Review Committee of The First Affiliated Hospital of China Medical University. (Ethical Review Code: AF-SOP-07-1.1-01).

### Survey scales

#### Geriatric depression scale (GDS)

The GDS [12] is a 30-item self-report assessment used to identify depression among the elderly. In the GDS, 20 questions are answered “yes” (scored 1) or “no” (scored 0), while the remain 10 questions are evaluated the opposite way (where “yes” is scored 0 and “no” is scored 1). The total score of the GDS ranges from 0 to 30, with a score of ≥11 indicating depression.

#### Pittsburgh sleep quality index (PSQI)

The PSQI [13] is a self-report questionnaire that assesses sleep quality over a one-month time interval. The measure scale consists of seven components, including subjective sleep quality, sleep latency, sleep duration, habitual sleep efficiency, sleep disturbance, use of sleeping medication, and daytime dysfunction. The score for each component ranges from 0 to 3, where 0 indicates “no difficulty” and 3 indicates “very difficult.” The total score of the PSQI ranges from 0 to 21, with a score of > 7 denoting poor sleep quality or having a sleep disorder.

Both scales are already used in China with citation. We also used a self-designed questionnaire for baseline information including age, sex, marital status, pension, economic status, chronic disease, self-care, body aches, chewing ability, ability to go out alone, exercise frequency, regular diet, smoking, filial piety, and loneliness. The researchers used self-assessment questionnaire to evaluate whether the participants were lonely or lack of family car.

### Quality control

We recruited 35 investigators to support the project and standardized training were provided for them in order to conduct questionnaire on their own. In consideration of the facts that some elderly people were already unable to read, we apply face-to-face interviews. During the interview, the investigator could inform the elder regarding the questionnaire in detail but could not be subjective nor changing the contents randomly. For certain cases, contents could be explained in more details on the condition that not affecting the survey result. Investigators observed participants’cognitive function to ensure the survey result accuracy.

### Statistical analysis

Items for which over 95% of participants had the same answers were excluded from the data analysis; these items included race (“Han” accounted for 96.8%) and medical insurance (“Have” accounted for 98.9%).

In addition, the proportion of missing items was < 5% among elderly adults with poor or good sleep quality. No missing items were processed. We performed a statistical analysis of the data using SPSS Statistics 20.0. We conducted a univariate analysis using the chi-square test, and a multivariate analysis using binary unconditional logistic regression analysis. A *P* value less than 0.05 was considered statistically significant. In this study, the elderly people with good sleep quality and poor sleep quality were investigated. The elderly depression in pension institutions was taken as the dependent variable, and the content in the general data was used as the independent variable.

## Results

### Sample characteristics

Among the 507 eligible participants, 253 had good sleep quality (71.6 ± 5.5 years old). This group comprised 125 females (49.9%) and 128 males (50.6%). Among them, 75 persons (29.6%) were aged < 75 years and 178 persons (70.4%) were aged ≥75 years. Furthermore, 235 elderly adults (92.9%) were married/cohabiting and 18 (7.1%) were divorced/widowed/unmarried. Two hundred and six (81.4%) participants had pensions and 47 (18.6%) did not. On the other hand, 254 elderly adults had poor sleep quality (71.9 ± 4.8 years old). This group included 129 females (50.8%) and 125 males (49.2%). Among them, 61 participants (24.0%) were aged < 75 years and 193 (86.0%) were aged > 75 years. Further, 194 participants (76.4%) were married/cohabiting and 60 (23.6%) were divorced/widowed/unmarried. One hundred eighty-five participants (72.8%) had pensions and 69 (27.2%) did not. The overall prevalence of depression was 21.7%; the ratios among elderly adults with good and poor sleep quality were 12.3 and 31.1%, respectively. The results of the chi-square test showed a significant difference in the depression rate between the two groups of elderly adults (*P* < 0.05).

### Univariate analysis of risk factors of depression according to sleep quality among elderly adults living in pension institutions

The results of the univariate analysis of factors influencing depression among elderly adults living in pension institutions are reported in Table 2. For elderly adults with good sleep quality, marital status, pension, economic status, chronic illness, self-care, body aches, chewing ability, ability to go out alone, regular exercise, eating patterns, smoking, and filial piety were significantly related to depression (*P* < 0.05). As for elderly adults with poor sleep quality, marital status, pension, chronic illness, self-care, chewing ability, ability to go out alone, regular exercise, diet, and loneliness were related to the detection rate of depression (*P* < 0.05).

**Table 2:** Univariate analysis of the factors influencing depression among elderly adults living in pension institutions

Factors		Total			Good sleep quality			Poor sleep quality		
		*n*	Depression Prevalence (%)	OR (95%CI)	*n*	Depression Prevalence (%)	OR (95%CI)	*n*	Depression Prevalence (%)	OR (95%CI)
Age	≧75 years	371	25.0	1.29 (0.81, 2.06)	178	13.3	1.15 (0.51, 2.58)	193	28.5	1.63 (0.89, 2.97)
	60–74 years	136	20.5		75	11.8		61	39.3	
Sex	Female	254	24.0	1.38 (0.90, 2.11)	125	15.2	1.73 (0.80, 3.74)	129	33.3	1.24 (0.73, 2.11)
	Male	253	19.0		128	9.4		125	28.8	
Marital status	Divorced/widowed/unmarried	78	56.4	7.12 (4.24, 11.96)	18	55.6	12.74 (4.54, 35.76)	60	56.7	4.33 (2.35, 7.97)
	Married/cohabiting	429	15.4		235	8.9		194	23.2	
Pension	No	116	37.9	3.01 (1.90, 4.76)	47	29.8	4.72 (2.12, 10.48)	69	43.5	2.14 (1.20, 3.80)
	Yes	391	16.9		206	8.3		185	26.5	
Economic status	Bad	52	34.6	2.08 (1.13, 3.86)	34	32.4	4.76 (2.03, 1 1.17)	18	38.9	
	Good	454	20.3		219	9.1		235	30.6	
Chronic disease	Yes	283	35.0	10.42 (5.42, 20.03)	114	24.6	14.76 (4.35, 50.04)	169	42.0	6.97 (3.17, 15.36)
	No	224	4.9		139	2.2		85	9.4	
Self-care	No	297	34.0	11.51 (5.66, 23.40)	134	20.9	10.21 (3.01, 34.58)	163	44.8	11.49 (4.75, 27.80)
	Yes	210	4.3		119	2.5		91	6.6	
Body aches	Yes	68	38.2	2.61 (1.51, 4.49)	34	32.4	4.76 (2.03, 11.17)	34	44.1	
	No	438	19.2		219	9.1		219	29.2	
Chewing ability	Poor	224	38.8	7.18 (4.34, 11.88)	100	21.0	3.80 (1.71, 8.47)	124	53.2	10.24 (5.23, 20.07)
	Good	283	8.1		153	6.5		130	10.0	
Ability to go out alone	No	186	37.1	4.03 (2.59, 6.27)	75	28.0	6.53 (2.90, 14.73)	111	43.2	2.75 (1.59, 4.76)
	Yes	321	12.8		178	5.6		143	21.7	
Regular Exercise	No	281	33.8	7.19 (4.03, 12.82)	105	24.8	9.41 (3.48, 25.48)	176	39.2	4.39 (2.11, 9.10)
	Yes	226	6.6		148	3.4		78	12.8	
Regular diet	No	201	35.3	3.74 (2.40, 5.83)	71	19.7	2.38 (1.11, 5.14)	130	43.8	3.62 (2.03, 6.44)
	Yes	306	12.7		182	9.3		124	17.7	
Smoking	Yes	145	22.1		73	5.5	3.04 (1.03, 9.04)	182	28.0	
	No	362	21.5		180	15.0		72	38.9	
Filial piety	No	125	28.0	1.59 (1.00, 2.53)	79	22.8	3.65 (1.69, 7.91)	46	37.0	
	Yes	382	19.6		174	7.5		208	29.8	
Loneliness	Yes	130	43.1	5.78 (2.40, 13.91)	42	17.6		88	59.6	11.10 (5.79, 21.27)
	No	377	20.0		211	19.4		296	21.6	

*CI* confidence interval, *OR* Odds ratio

### Multivariate analysis of risk factors of depression according to sleep quality among elderly adults living in pension institutions

The results of the multivariate analysis of risk factors for depression among elderly adults with different sleep quality living in pension institutions are reported in Table 3. After adjusting for age as a fixed factor in the model, the logistic regression analysis revealed that the factors influencing depression among elderly adults with good sleep quality were ranked (in descending order of effect size) as follows: marital status, chronic disease, regular exercise, body aches, child filial piety, and chewing ability (*P* < 0.05). By contrast, the factors influencing depression among elderly adults with poor sleep quality were ranked as follows: loneliness, regular diet, chewing ability, and chronic disease (*P* < 0.05; Table 3).

**Table 3:** Multivariate analysis of factors influencing depression among elderly adults with different sleep quality living in pension institutions

Factor	OR	95% confidence interval	*P*
Good sleep quality (*n* = 253)			
Age^a^	1.12	1.00–1.24	0.04
Marital status (divorced/widowed/unmarried vs. married/cohabiting)	6.91	1.79–26.79	< 0.01
Chronic disease (Yes vs. No)	6.66	1.64–26.96	< 0.01
Regular exercise (No vs. Yes)	5.11	1.56–16.67	< 0.01
Body aches (Yes vs. No)	4.10	1.30–12.97	0.02
Filial piety (No vs. Yes)	3.79	1.34–10.73	0.01
Chewing ability (Yes vs. No)	2.74	1.00–7.51	0.05
Poor sleep quality (*n* = 254)			
Age^a^	0.96	0.89–1.04	0.32
Loneliness (Yes vs. No)	8.77	2.37–32.54	< 0.01
Self-care (No vs. Yes)	7.00	2.25–21.75	< 0.01
Chewing ability (Yes vs. No)	5.31	2.47–11.40	< 0.01
Chronic disease (Yes vs. No)	4.40	1.64–11.83	< 0.01

^a^Fixed in the model; age was set as a continuous variable in the multivariate analysis

## Discussion

This study conducted a cognitive function screening for elderly adults living in pension institutions, and adopted the highly valid and reliable GDS as the main assessment tool for evaluating depression and its influencing factors among elderly adults without cognitive dysfunction. The findings of this study can be characterized as a good representation of the population (and thus bearing high information reliability). The conclusions drawn also have strong generalizability. The results of this study showed that the depression rate among elderly adults without cognitive dysfunction living in pension institutions in Northeastern China was 21.7%, which is much lower than that in Beijing (*n* = 107, 32.71%) [14] and Guangdong Province (*n* = 379, 38.5%) [15]. The depression rate among elderly adults with poor sleep quality was 31.1%, which was significantly higher than that among elderly adults with good sleep quality (12.3%). The results were similar to those pertaining to elderly adults living in pension institutions in Beijing [14].

Similarly, Dai and Li et al. found a significant negative correlation between sleep quality and depression [5, 14]. However, there was no study to discuss whether there were differences in depression influencing factors among the elderly with different sleep quality. This study hopes to further analyze the influencing factors of depression in the elderly with different sleep quality and provide more specific measures. The following will reveal accordingly based on sleeping quality.

For the elderly with good sleep quality, spousal support and care can reduce the incidence of depression: indeed, the incidence of depression in widowed elderly adults was significantly higher than that among married elderly adults [16]. Divorced elderly adults or those in poor marriages are also more likely to suffer from mental health problems and increased depression [17]. However, one study has shown that there was no relationship between depression and marital status [18]. It may be because the nature of the pension institution is different from that of home-based pension. Even if the elderly (in pension institution) has a spouse, they cannot be accompanied for a long time.

Regular exercise can strengthen the body and increase the opportunity to communicate with others while exercising, thereby sustaining feelings of pleasure among elderly adults [19]. However, there are some risks in physical exercise in the pension institution [20]. At present, there were also control experiments of physical exercise in the pension institutions. Although the conclusion shows that exercise can alleviate the depression of the elderly. But the sample size of the study was small and the exercise mode was only a single Taiji exercise. It can not prove the safety and effectiveness of physical exercise [21]. Therefore, this conclusion needs to be treated with caution. The implementation of physical exercise in the pension institutions requires the supervision and guidance of a professional rehabilitation team, which most of the pension institutions are lack of. In this study, various physical exercise for elder in pension institutions are carried, which include table tennis, running, dancing and etc. In the future, with the guidance of professional teams, we could carry out research focusing one form of sports.

Elderly adults who often experienced body aches had a higher risk of depression. Body aches are likely to increase discomfort among elderly adults, which in turn can affect their daily activities and increase their negative emotions. Some studies show that there is a relationship between the degree of physical pain and depression in the elderly [22]. However, others are indicating no correlation in such relationship as high tolerance of pain, reduced sensitivity to pain, and elderly people subjectively thinking the pain is the natural result of the age could also be the possibilities [23]. Nevertheless. It is essential to pay attention to the physical pain of the elderly. Meanwhile, we should encourage the elderly to honest about the pain level so that medical personnel could provide corresponding measures to release the pain. Painkiller could be provided according to the standard. Taking medication without medical advice creates risk at the same time. There are adverse reactions in the elderly after self medication [24]. We cannot encourage the elderly to use drugs blindly without the professional guidance of doctors in order to alleviate the pain. Therefore, this conclusion needs more detailed verification.

For elderly adults with poor sleep quality, loneliness is an important risk factor of depression. Insomnia can increase elderly adults’ sense of loneliness, which is likely to cause depression [14]. Similarly, chewing ability has an impact on depression among elderly adults with poor sleep quality. Therefore, we should pay close attention to the chewing ability of elderly adults. Pension institutions should provide soft food to elderly adults based on their chewing ability. These institutions should also conduct regular physical examinations of elderly adults, supervise the rational and timely use of drugs among elderly adults with chronic diseases, and formulate measures to reduce disease-related psychological burdens for elderly adults, thereby reducing the incidence of depression among elderly adults living in pension institutions. Under the present conditions in China, the majority elders choose to live at home together with their spouse or children. Only a small portion of elder Chinese are living in pension institutions. With no massive markets, pension institutions in China lack of systemic management and professional nursing staff. There is also a lack of professional guidance regarding elderly depression treatment. In such case, we need to make continuous contributions to further develop comprehensive pension instituions.

There were some limitations in our study. First, measures for some of the factors, such as smoking and chewing ability, were simplistic and broad, and therefore could have weakened our assessments of their effects. Second, although the present study was a population-based study on depression among elderly adults with different sleep quality in pension institutions in Northeastern China, it was limited by its cross-sectional design. Third, loneliness was not evaluated by the scale in the present study, and will be considered in the future. Therefore, all conclusions drawn from this study should be confirmed in a future prospective study.

In conclusion, this study is the first to assess depression among elderly adults with different sleep quality in pension institutions in Northeastern China and to clarify its associated factors. Our results revealed that the prevalence of depression differentiated among elderly adults according to their sleep quality. Marital status, chronic disease, regular exercise, body aches, filial piety, and chewing ability had considerable effects on depression among elderly adults with good sleep quality. By contrast, loneliness, self-care, chewing ability, and chronic disease had considerable effects on depression among elderly adults with poor sleep quality. Our findings suggest that there should be a focus on the maintenance of health status and psychological factors to reduce the incidence of depression among elderly adults in pension institutions.

## Data Availability

The datasets used and analyzed during the current study are available from the corresponding author on reasonable request.

## Abbreviations

GDS: Geriatric Depression Scale
MMSE: Mini-Mental State Examination
PSQI: Sleep Quality Index
